# Editorial: Canine Hip and Elbow Dysplasia Improvement Programs Around the World: Success or Failure?

**DOI:** 10.3389/fvets.2021.713042

**Published:** 2021-08-31

**Authors:** Mário Ginja, José Manuel Gonzalo-Orden, António Ferreira

**Affiliations:** ^1^Department of Veterinary Science, University of Trás-os-Montes and Alto Douro, Vila Real, Portugal; ^2^CITAB—Centre for the Research and Technology of Agro-Environmental and Biological Sciences, Vila Real, Portugal; ^3^CECAV—Veterinary and Animal Research Centre, Vila Real, Portugal; ^4^Department of Veterinary Medicine, Surgery and Anatomy, Faculty of Veterinary Medicine, University of León, León, Spain; ^5^CIISA—Department of Clinics, University of Lisbon, Lisbon, Portugal

**Keywords:** hip dysplasia, elbow dysplasia, breeding value, genomic selection, hip laxity

This compilation of 13 papers under the Research Topic “Canine Hip and Elbow Dysplasia Improvement Programs Around the World: Success or Failure?” addresses different aspects of the research that is currently being done about canine hip dysplasia (HD) and elbow dysplasia (ED), giving a special emphasis to the analysis of HD and ED in some canine populations over time. We can say that hip HD first, and then ED have been responsible for a real headache for veterinarians and dog owners for the past 50 years and an important limitation on the welfare of affected dogs. Both diseases continue to have a high prevalence, especially in some large and giant canine breeds.

Recognition of hereditary HD etiology coincides with the first descriptions of the disease. However, knowledge in the form of inheritance has evolved from a simple Mendelian inheritance to a polygenic and multifactorial nature.

The strategy to deal with canine HD and ED was based on two essential pillars, with the aim, on the one hand, of selecting animals without the disease for breeding to reduce the frequency of bad genes in the population, and, on the other, of treating the individual affected animal to improve their well-being. In this aspect, radiographic examination has been used since the 1960's for HD diagnosis and for selection of animals for breeding with the aim of eradicating the disease (1). The diagnosis of HD on radiographs has been grounded in the detection of signs of degenerative joint disease. These aspects were well-highlighted in the reviews by Hedhammar and Anderson et al. presented in this collection of papers. In these early days, the selection of animals for breeding was based on the individual phenotype and the main aims were to reduce/eradicate HD prevalence. Many control programs fell rather short of what was expected, which led to some disbelief, mainly on the part of the breeders (2). Dogs with a normal phenotype may nevertheless be carriers of genes related to hip dysplasia. Since the 1980's, HD radiographic diagnostic methods based on passive hip laxity have also been developed. Hip laxity (HL) is considered the major HD risk factor. Hip laxity allows us to know the predisposition for HD earlier, at 4 months of age, the examination requires the use of a hip distractor. The use of HL is more popular in the United States, but recently two hip distractors have appeared in Europe, the Vezzoni and the DisUTAD. The latter is presented in this collection by Santana et al. (a), and it is currently being used in Portugal and Spain, and it is expected that it will become more widely used.

Most HD and ED radiographs are obtained with the examiner's physical restraint of the dog on the X-ray table. However, precautions must always be taken to reduce the potential human exposure to the harmful effects of ionizing radiation. In this collection, Santana et al. (b) Present a paper in which HD radiographs were obtained using a hindlimb holder device, which allows HD images of high technical quality without any examiner help and without human exposure to ionizing radiation within the x-ray room.

A recent reality in terms of radiographic images was the introduction of the digital image. On the one hand, it can be an asset in the correction and optimization of some lower-quality images resulting from inappropriate x-ray beam exposure factors. But on the other hand, they are also subject to specific quality aspects that, if not properly understood, may result in inadequate radiographic diagnoses. Some of these aspects are well-illustrated in relation to HD and ED in this collection in papers by Moorman et al. and Válega et al. respectively.

Elbow dysplasia is especially important in the dog, while HD is also clinically very important in humans. The strategy with respect to human HD has always been focused on diagnosis and treatment, and this has been a great success, both in respect to disease prevention and to treatment. In veterinary medicine, the success is also very satisfactory in terms of therapeutic options for advanced disease with joint destruction, where we highlight the success of treatments with complete total hip replacement (3). For the elbow joint, the total replacement option is not so common. Some basic research in this area is still needed. Aiyan et al. present a paper to study the proximal femoral morphology. For severe HD or ED cases, other therapeutic options, some surgical and others non-surgical, that are more conservative and less expensive remain available. In the papers by Silva Júnior et al. and Kriston-Pál et al., the administration of intra-articular substances is described. However, there is clearly a gap in veterinary medicine; it is the absence of a truly early diagnosis and of preventative complementary management methods that both reduce the severity of HD, and are easily accessible to all dogs, even mild forms of the disease. We believe that mild forms of disease, even if dogs do not show evident clinical signs, may be associated with discomfort and reduced animal welfare. In this regard, the approach to HD management in terms of human medicine is clearly more developed. In humans, the diagnosis of developmental dysplasia is confirmed at birth, since in this case HD has the peculiarity of being congenital, and preventive management is recommended for all situations. As for the early diagnosis of HD in dogs, it usually takes place at about 4 months of age and preventive treatment is usually reserved for animals with clinical signs of the disease, we think that there are great possibilities for an optimization approach here. Furthermore, current surgical treatments in canine HD involve altering the normal pelvic bone structure of the animal, and the response to the most severe forms of the disease is not always the best.

Based on the evolution of knowledge on the control of other inherited polygenic and environmental characteristics, such as the production of milk and meat in farm animals, the control of HD and ED in dogs was redirected. The reference ceased to be the individual phenotype and became the breeding value. The selection of dogs by HD breeding values requires HD databases with the largest number of animals possible. Breeding values are a more reliable reference of the genetic component of the animals for the disease, and the current results are considered very promising; these aspects are well-documented in this collection in the papers of Wang et al., and James et al. Therefore, the current recommendation for the control of hip and elbow dysplasia is to use breeding values as the main selection criteria. Some studies to better understand the form of HD and ED heritability continue to be carried out; Baers et al. presents a paper on this subject. However, the use of individual phenotypes for animal selection in control programs continues to be used in some countries with success in reducing prevalence and severity in canine populations (Ohlerth et al.). We think that in this area, there is now sufficient knowledge and means to deal with HD and ED diseases effectively.

However, currently the goal is the evolution toward genomic selection, which is already a present reality in farm animal milk and meat production and allows animal selection without the need for phenotype population databases. Genomic selection has been based on the detection of single nucleotide polymorphisms associated with genetic characteristics. Hip dysplasia is also taking the first steps in this direction. A molecular test has already been made available, the Dysgen, although it has not achieved the desired success (4). An obvious advantage of this approach in the future is that there is no need for a radiographic examination and all associated medical procedures.

In response to the question of the topic, we believe that over these 50 years the successes and advances made in the knowledge of HD and ED have been very significant and have undoubtedly eventually overcome failures that may have existed. It is evident that there has been an evolution in the strategies that have been used over time to deal with HD and ED, both in terms of treatment and in the selection of animals for breeding. But it is also notable that in both aspects related to these health areas, additional improvements are possible based on more targeted research. We hope that this collection of research articles will serve to increase awareness of current activity in this area and point to possible potential improvements and developments in the fields of hip and elbow joint dysplasia.

## Author Contributions

All authors listed have made a substantial, direct and intellectual contribution to the work, and approved it for publication.

## Conflict of Interest

The authors declare that the research was conducted in the absence of any commercial or financial relationships that could be construed as a potential conflict of interest.

## Publisher's Note

All claims expressed in this article are solely those of the authors and do not necessarily represent those of their affiliated organizations, or those of the publisher, the editors and the reviewers. Any product that may be evaluated in this article, or claim that may be made by its manufacturer, is not guaranteed or endorsed by the publisher.
